# THE POTENTIAL USE OF INTRANASAL OXYTOCIN AS EARLY PREVENTIVE INTERVENTION FOR POST-TRAUMATIC STRESS DISORDER

**DOI:** 10.1192/j.eurpsy.2023.2084

**Published:** 2023-07-19

**Authors:** M. A. L. Magalhães, M. Andrade

**Affiliations:** Centro Hospitalar Psiquiatrico de Lisboa (CHPL), Lisboa, Portugal

## Abstract

**Introduction:**

Post-traumatic stress disorder (PTSD) is defined by an exaggerated fear responses (FA) which fails to extinguish over time and cannot be inhibited in safe contexts. Studies report that traumatic experiences (TE) affect hormonal systems mediated by the hypothalamic-pituitary-adrenal (HPA) axis and the oxytocinergic system. Oxytocin (OXT) is a neurohormone produced in the hypothalamus that has social functions like the promotion of prosocial and affiliative behaviors, increased self-confidence and positive social memories. In PTSD there is a diminished inhibitory top-down control over the FA, which is characterized by amygdala hyperactivity, ventromedial prefrontal cortex (vmPFC) hypoactivity and diminished structural and functional connectivity between both areas, which results in anxiety increase and dysregulated autonomic and endocrine FA. In parallel, TE decrease the synthesis and release of OXT, resulting in the dysfunction of the negative feedback mechanism on the HPA, leading to hypercortisolemia and maximizing the response to a stressful stimulus. Previous studies report that the administration of OXT can reduce cortisol levels as well as attenuating amygdala hyperactivity and normalizing the connectivity of this structure with frontal areas, diminishing the FA. Therefore, OXT has been investigated as a potential therapeutic agent administered intranasally early after trauma as a strategy to prevent PTDS on individuals having high risk.

**Objectives:**

The aim of this work is to review the potential of intranasal OXT administration as early preventive intervention for PTSD.

**Methods:**

Systematic review of the literature published in Pubmed, using the terms “Oxytocin”, “Post-traumatic Stress Disorder”, “Stress”.

**Results:**

Studies found significant associations between TE and OXT and report that TE and PTSD are strongly associated with reductions in OXT. Literature report that the acute effects of OXT administrations in individuals with TE tend to be anxiolytic only in less severe forms, by modulating the HPA axis and the autonomic nervous system. Moreover, in recent TE, OXT seems to increase the re-experience of traumas and restore the function of different networks associated with fear control in PTSD patients. FMRI studies indicate that intranasal OXT attenuates amygdala hyperactivity and enhances amygdala´s connectivity with vmPFC, resulting in increased control over the FA. Finally, studies report that a single oxytocin administration increases neuronal fear processing but repeated administration reduces PTSD symptoms up to 6 months post trauma in patients with high acute symptoms.

**Conclusions:**

Repeated administration of intranasal oxytocin early after trauma seems to diminish the acute symptoms in early stages of PTDS, being a potential pharmacological strategy to prevent PTDS in individuals at high risk by increasing the control of FA.

**Disclosure of Interest:**

None Declared

